# Filled Carbon Nanotubes: Promising Material for Applications

**DOI:** 10.3390/nano13172472

**Published:** 2023-09-01

**Authors:** Marianna V. Kharlamova

**Affiliations:** 1 Faculty of Physics, University of Vienna, Strudlhofgasse 4, 1090 Vienna, Austria; mv.kharlamova@gmail.com; 2 Moscow Institute of Physics and Technology, National Research University, 9 Institutskiy Per., 141701 Dolgoprudny, Russia

Carbon nanotubes (CNTs) were first filled with a number of metals starting in 1993 [1,2]. Single-walled carbon nanotubes (SWCNTs) were first filled in 1998 by the group of Jeremy Sloan [3]. Filled carbon nanotubes represent a carbon nanostructure where an interior channel of CNTs incorporates a foreign substance. The filled CNTs are denoted as X@CNT. These nanostructures combine the unique properties of CNTs and foreign substances. Since then, the family of filled carbon nanotubes rose to about 200 nanostructures where CNTs introduce foreign substances. These can be simple substances, chemical compounds, or molecules. The two simple substances are metals and non-metals. The chemical compounds are metal halogenides of transition, lanthanide, alkali, p-metals, and metal chalcogenides. The molecules are metallocenes, metal acetylacetonates, and fullerenes.

Several groups are working on the topic. The groups of Jeremy Sloan in the UK [4,5], Marianna V. Kharlamova in Austria and Slovakia [6,7], Hisanori Shinohara in Japan [8,9], Antonio Setaro and Stephanie Reich in Germany [10,11], and Gerard Tobias in Spain [12,13]. 

First of all, more substances are waiting to be incorporated into carbon nanotubes. Secondly, the filling of carbon nanotubes opens new possibilities. Third, incorporated substances form new phases inside CNT. Many new works on modeling the atomic structure of filled SWCNTs are forthcoming. Fourth, the filling modifies the electronic properties of SWCNTs. In all cases, the collaboration of researchers around the world is required, which brings new ideas and results. 

The electronic properties of SWCNTs were first modified and extensively investigated by Marianna V. Kharlamova [14,15,16]. In this paper, the SWCNTs were filled with metal halogenides, and metal chalcogenides, and the electronic properties were studied by Raman spectroscopy, X-ray photoelectron spectroscopy, optical absorption spectroscopy, and near edge X-ray absorption fine structure spectroscopy. It was shown that intorporated substances lead to p-doping of SWCNTs. 

A large variety of incorporated substances allows tuning the electronic properties of SWCNTs for targeted applications. Recently, Hisanori Shinohara et al. filled SWCNTs with HgTe, WTe, and MoTe and investigated the electronic properties of filled SWCNTs with spectroscopic techniques [8,9]. It is nice that researchers develop the topic and become involved in the forthcoming boom of investigations into filled carbon nanotubes [17,18]. I think that this topic is a way from lab scale to industrial applications of SWCNTs. 

Speaking of applications, most carbon nanotube papers target nanoelectronics, electrochemical energy storage, nanomedicine, thermoelectric power generation, catalysis, sensors, spintronics, and magnetic recording. From one side, all these applications can be developed on an industrial scale. On the other side, the synthesis methods of filled SWCNTs, manipulation, and building device methods are optimized and ready to be implemented in factories. For these reasons, a boom in investigations of filled CNTs is expected. 

In the Special Issue entitled “Applications of Functionalized Carbon Nanomaterials: Advances and Perspectives”, we focus on applications of chemically functionalized carbon nanomaterials such as carbon nanotubes, graphene, graphene nanoribbons, 2D heterostructures, fullerenes, and nanodiamonds. 

In my review paper [19], I summarize the main achievements in metal halogenide, metal chalcogenide, metal, and metallocene-filled SWCNT research. The data from the analysis of the kinetics and electronic properties of filled SWCNTs were reviewed. New methods of processing the spectroscopic data were developed. The dependence of chemical properties on the chemical nature of substances was discussed. The achievements in metallicity-sorted SWCNT filling were described. The correlations between the chemical and physical properties of introduced substances and outer SWCNTs were highlighted. 

In a research paper [20], the authors studied in detail the applications of aminated graphene for the covalent conjugation of monoclonal antibodies. They applied X-ray photoelectron and absorption spectroscopies and electron microscopy to their investigations (Figure 1). Chemiresistive biosensors composed of aminated graphene and conjugated antibodies were made. The authors showed that there is a selective response towards IgM immunoglobulins with a low limit of detection (Figure 2). 

Interested authors are invited to submit their excellent experimental and theoretical works to this special issue entitled “Applications of Functionalized Carbon Nanomaterials: Advances and Perspectives”. 

## Figures and Tables

**Figure 1:**
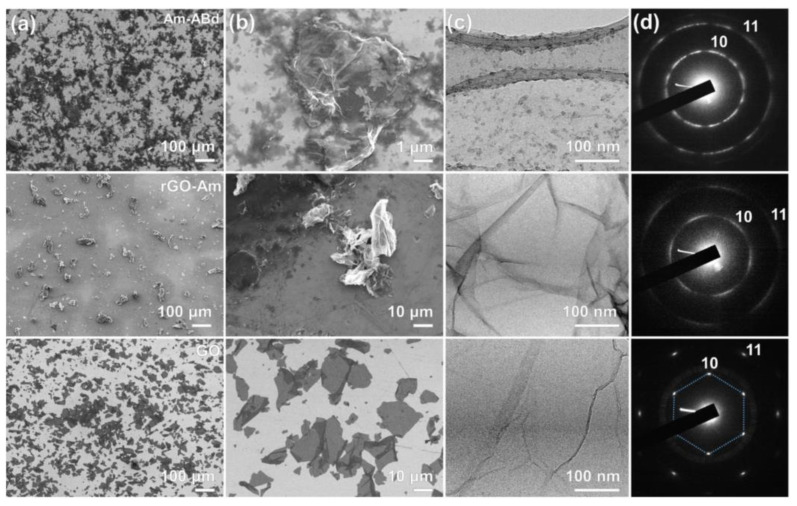
(**a**,**b**) Scanning electron microscopy images; (**c**) transmission electron microscopy images; and (**d**) electron diffraction patterns of the initial graphene oxide (**bottom row**), aminated graphene (rGO-Am) (**middle row**), and aminated graphene with the immobilized antibodies (Am-ABd) (**upper row**) layers. Copyright 2023 by the authors. Licensee: MDPI, Basel, Switzerland. This article is an open access article distributed under the terms and conditions of the Creative Commons Attribution (CC BY) license.

**Figure 2:**
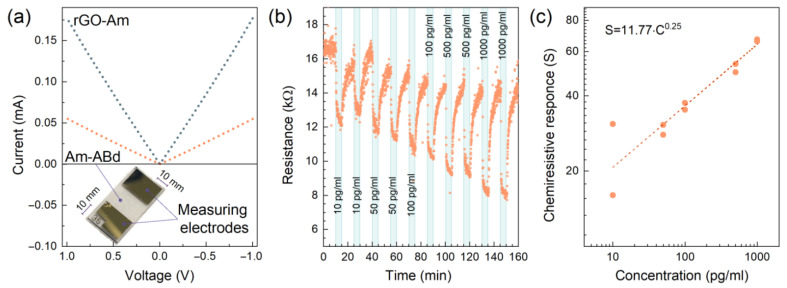
(**a**) I–V curves for the aminated graphene (rGO-Am) and aminated graphene with the immobilized antibodies (Am-ABd) layers. The insert shows a photo of the biosensor. (**b**) The resistance transient recorded under exposure to PBS solution with IgM immunoglobulins. (**c**) The chemiresistive response versus the concentration of the IgM immunoglobulins. Copyright 2023 by the authors. Licensee: MDPI, Basel, Switzerland. This article is an open access article distributed under the terms and conditions of the Creative Commons Attribution (CC BY) license.

## Data Availability

The data are available on request from the author.
